# Heterostructure growth, electrical transport and electronic structure of crystalline Dirac nodal arc semimetal PtSn_4_

**DOI:** 10.1038/s41598-024-81679-2

**Published:** 2024-12-28

**Authors:** Edward L. Beynon, Oliver J. Barker, Tim D. Veal, Liam O’Brien, Marita O’Sullivan

**Affiliations:** Department of Physics, University of Liverpool, Oxford Street, Liverpool, L69 7ZE UK

**Keywords:** Condensed-matter physics, Electronic properties and materials, Surfaces, interfaces and thin films

## Abstract

Topological semimetals have recently garnered widespread interest in the quantum materials research community due to their symmetry-protected surface states with dissipationless transport which have potential applications in next-generation low-power electronic devices. One such material, $\hbox {PtSn}_{4}$, exhibits Dirac nodal arcs and although the topological properties of single crystals have been investigated, there have been no reports in crystalline thin film geometry. We examined the growth of $\hbox {PtSn}_{4}$ heterostructures on a range of single crystals by optimizing the electron beam evaporation of Pt and Sn and studied the effect of vacuum thermal annealing on phase and crystallinity. The electrical resistivity was fitted to a modified Bloch–Grüneisen model with a residual resistivity of 79.43(1) $\mu \Omega$cm at 2K and a Debye temperature of 200K. Nonlinear Hall resistance indicated the presence of more than one carrier type with an effective carrier mobility of 33.6 $\hbox {cm}^2\, \hbox {V}^{-1}\, \hbox {s}^{-1}$ and concentration of 1.41 $\times 10^{21}\, \hbox {cm}^{-3}$ at 300 K. X-ray photoemission spectra were in close agreement with convolved density of states and a work function of 4.7(2) eV was determined for the $\hbox {PtSn}_{4}$ (010) surface. This study will facilitate measurements that require heterostructure geometry, such as spin and topological Hall effect, and will facilitate potential device incorporation in future quantum technologies.

## Introduction

Topological quantum materials can exhibit conducting surface states which are protected from losses by symmetry and have potential applications in future energy-efficient computing technology for data storage^1^. A major focus of materials research since the Nobel prize in 2016 was awarded for topological crystalline materials has been the search for topological insulators^2^ with heavy metal compositions to introduce spin-orbit coupling which opens a band gap across the Fermi level. Semimetals differ from their insulator counterparts in that the effective valence and conduction bands both cross the Fermi level, usually at different points in reciprocal space, rendering them indirectly gapless, such that both holes and electrons contribute to the electrical conductivity. In topological semimetals the bands touch at nodal points or lines in momentum space eliciting conducting surface states which are robust to dissipation^3–5^. Intermetallics composed of transition metals of the 5d series, possessing the spin-orbit interaction associated with topological quantum materials, may have the potential to demonstrate these line nodes which are predicted to form closed loops over momentum space^6^ in 3D systems.

$\hbox {PtSn}_{4}$ is a promising semimetallic intermetallic system for the study of Dirac fermions which exhibits Dirac nodal arcs where the valence and conduction bands cross each other such that they do not complete a closed loop in momentum space, but rather have short line segments arising similar to the surface states of graphene^7^. Researchers have investigated the topological^1^ and magnetotransport properties^8^ and the electronic structure in bulk and single crystals of $\hbox {PtSn}_{4}$ using angle-resolved photoemission spectroscopy and compared it to density functional theory (DFT) calculations of the surface band structure^7^. Single crystals of this material are reported to exhibit an extremely high magnetoresistance of up to 5$\times 10^5$% which is a signature of topological semimetals for the highest quality crystals^8,9^ and the planar Hall effect measurements point to a chiral anomaly^10^. The magneto-thermoelectric properties of this intermetallic semimetal have been investigated^11^. The de Haas-van Alphen frequencies of $\hbox {PtSn}_{4}$ single crystals have been studied at high magnetic fields and compared to the calculated complex Fermi surface^8,12,13^ with several hole and electron pockets which are similar in size. $\hbox {PtSn}_{4}$ has applications as a replacement for Pt in catalysis^14–16^ and the linear band crossings provide enhanced carrier mobility and conductivity of the massless Dirac fermions to promote this process^17^.

While a considerable research effort has been devoted to the study of bulk and single crystal $\hbox {PtSn}_{4}$, this system is less studied in heterostructure geometry, which would facilitate its integration in future spintronic devices, and the reported films are amorphous^18^. Thin films of $\hbox {PtSn}_{4}$ have been grown in a CoFeB bilayer as an amorphous layer with a large magnetoresistance^18^, and while a number of studies of the wetting of molten Sn and Sn-based solder at the Pt interface have been conducted^19,20^, crystalline thin films have not yet been realized. Metallic non-oxide thin films are typically grown on either single crystal Si wafers or glass substrates as geometrical considerations such as coincident-site lattice matching and epitaxial strain have less dramatic effects on the physical properties than they do in the more localized oxide systems. Growth of semiconductor/dielectric interfaces has only recently received attention from the research community due to the dielectric applications of oxides in metal oxide semiconductor field effect transistors^21^. The use of oxide single crystals to promote the growth of crystalline and epitaxial metallic non-oxide thin films is a relatively unstudied area of materials science and could have a positive impact on the study of these topological materials in a more application-amenable architecture. Single crystals of $\hbox {PtSn}_{4}$ have previously been synthesized by flux growth from the metallic melts^22^, however thin film growth is complicated by the presence of Sn, a volatile metal which oxidizes readily upon exposure to oxygen atmosphere at elevated temperatures. It was therefore important to deposit this material in a reducing environment and to carry out any post-deposition annealing steps under vacuum. Growth of intermetallic thin films could be achieved using a number of methods. In this work we selected electron beam evaporation of Pt and Sn metal sources. We investigated the importance of substrate selection, we examined the techniques of Pt/Sn bilayer deposition and co-deposition of the constituent elements and we studied the effects of post-deposition vacuum annealing on the sample crystallinity.

## Methods

Electron beam evaporation was used to grow the films by bombarding elemental Pt and Sn source metals with electrons in an ultra-high vacuum chamber with a base pressure of 5$\times 10^{-10}$ mbar. This technique was selected over other deposition techniques due to the extremely high melting temperature of Pt. The binary phase diagram indicates that the $\hbox {PtSn}_{4}$ phase forms on a peritectic and is stabilized at temperatures up to $540^{\circ }$C for a 4:1 atomic ratio of Sn:Pt^23–25^. A quartz crystal microbalance was used to monitor the growth rate of each of the metals. The methods of co-deposition and bilayer deposition were explored to study the growth mode of the films. The source metals had purities of 99.998% for Sn and 99.99% for Pt. The growth pressure for the co-deposition was 2.1 $\times \, 10^{-7}$ mbar and for the bilayer depositions the growth pressure for Sn was 2 $\times \, 10^{-8}$ mbar and for Pt it was 2 $\times \, 10^{-7}$ mbar. The co-deposited samples with a target thickness of 30 nm were grown with a 1:4 atomic ratio of Pt:Sn to achieve the $\hbox {PtSn}_{4}$ stoichiometry. The bilayer samples involved deposition of the component elements in two stages, the initial Sn layer was followed by the Pt layer with a thickness monitored using the quartz microbalance, giving an atomic ratio of 4:1 to achieve the targeted composition. The substrates were not heated however the evaporated material arrived at the surface with sufficient kinetic energy to mobilize the ions within the growing layer. The samples were also subjected to post-deposition vacuum annealing at temperatures up to $500 \,^{\circ }$C to examine the effect of redistributing the metal ions in the bilayer depositions and recrystallizing the grains of both the bilayer and co-deposited films. The annealing was carried out in a vacuum chamber with a base pressure of 1$\times 10^{-6}$ mbar using a graphite resistive heater with heating and cooling at a rate of 10–25 $^{\circ }$C/min and a 60 min dwell time at the set temperature with thermocouple monitoring.

The thickness, roughness, phase, orientation and crystallinity of the deposited layers were assessed using X-ray scattering techniques including X-ray reflectivity and X-ray diffraction which were carried out on a high-resolution ($0.0002^{\circ }$) four-circle Rigaku Smartlab X-ray diffractometer with 9 kW rotating anode Cu $K_{\alpha 1}$ radiation ($\lambda$ = 1.540593 Å). The parallel beam geometry was used with a 2 bounce Ge (220) monochromator and a HyPix-3000 2D detector in 1D mode.

Hall bars were patterned on the 30 nm thick $\hbox {PtSn}_{4}$ thin film samples by shadow masking during the deposition and the electrical and magnetotransport properties, including resistivity, magnetoresistance and Hall effect were measured in this configuration to extract the transport parameters. The films deposited using shadow masking present the same diffraction pattern to the films deposited with no mask (Fig. S1). The measurements were carried out in a 14 T Quantum Design Dynacool Physical Property Measurement System with the Electrical Transport Option using In soldered contacts and Au wire with an AC excitation amplitude of 0.1 mA and a frequency of 18.31055 Hz. The resistivity was measured as a function of temperature from room temperature down to 2 K. The symmetric component of the resistivity tensor as a function of magnetic field was used to determine the magnetoresistance of the material. The antisymmetric component was used to extract the transverse Hall resistance for an indication of the number of carriers, the carrier types and for the calculation of the effective carrier concentration and effective carrier mobility.

The electronic structure of the heterostructures was investigated by X-ray photoemission spectroscopy (XPS) in an ultra-high vacuum system (3$\times10^{-11}$ mbar) with an Omicron SPHERA electrostatic hemispherical deflection analyser with a mean radius of 125mm and a monochromated Al $K_{\alpha 1}$ photon source (h$\nu$ = 1486.6 eV). Photoelectron spectra were collected corresponding to the Pt 4*f* and Sn 3*d* levels. The instrumental energy resolution was 0.4 eV and the binding energy was calibrated from a polycrystalline metallic silver reference standard.

**Figure 1:**
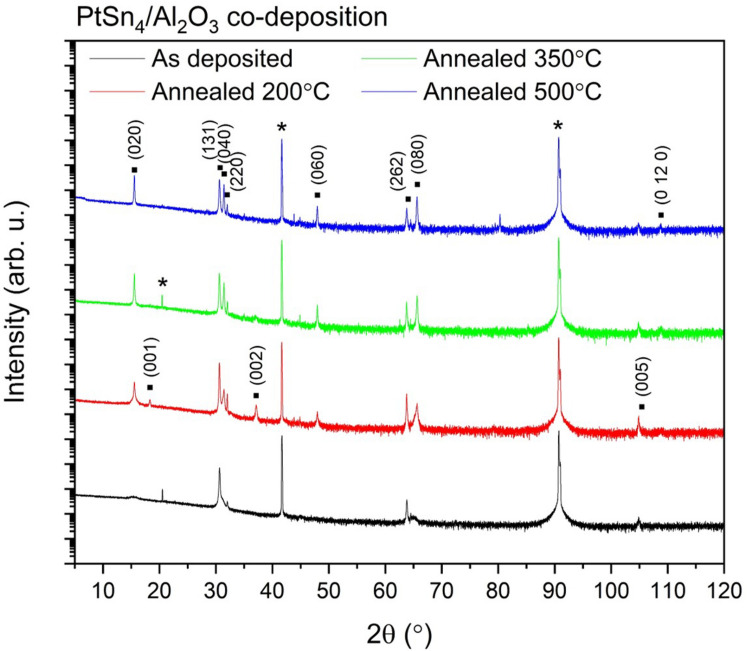
2$\theta$ X-ray diffraction scans of co-deposited $\hbox {PtSn}_{4}$ thin films grown on (0001) oriented $\hbox {Al}_{2}\hbox {O}_{3}$ substrates. The graph shows the diffraction pattern of the as-deposited film (black), together with those of the films which were vacuum annealed at $200^{\circ }$ (red), $350^{\circ }$ (green) and $500^{\circ }$ (blue). The observed Bragg reflections are labelled on the diffractograms and (*) indicates substrate peaks.

Density functional theory calculations were carried out using the Vienna Ab initio Simulation Package (VASP)^26^ with the plane-wave basis of the projector augmented wave method^27^. The generalized gradient approximation was used with the Perdew-Burke-Ernzerhof functional correction to the exchange correlation with a plane-wave cutoff of 400 eV^28^. The spin-orbit interaction was included in the calculations in a gamma-centered 12 $\times$ 8 $\times$ 12 k-point mesh. The valence edge of the photoemission spectra of the samples was compared to the calculated partial densities of states using Galore software^29^ with an instrumental broadening of 0.4 eV and a lifetime broadening of 0.5 eV.

## Results and discussion

The study of the optimum growth conditions for $\hbox {PtSn}_{4}$ thin films deposited by electron beam evaporation involved the investigation of a range of single crystal substrates with different structures distinct from that of the film, including (100) oriented Si, (110) and (111) oriented $\hbox {SrTiO}_{3}$ and (0001) oriented $\hbox {Al}_{2}\hbox {O}_{3}$. It is difficult to establish epitaxial growth at the interface between a metal or semiconductor and an oxide as evidenced by the many studies of $\hbox {SrTiO}_{3}$ on Si^21^. This is due in part to the geometrical mismatch of the crystal structures, including different lattice parameters and discontinuous packing and stacking of the atoms, but also to the discontinuous bonding at the interface. X-ray diffraction patterns for the as-deposited samples on the $\hbox {SrTiO}_{3}$ and $\hbox {Al}_{2}\hbox {O}_{3}$ substrates yielded single phase, crystalline films of $\hbox {PtSn}_{4}$ for both the co-deposited and bilayer deposited samples. This indicates that electron beam evaporation is a suitable technique for the deposition of crystalline intermetallic thin films with this composition. The samples grown on Si (100), however, were amorphous.

### Heterostructure growth and structural properties

The co-deposited $\hbox {PtSn}_{4}$ films were deposited on c-cut sapphire substrates and XRD 2$\theta$ scans shown in Fig. 1 (black) indicated that these films were preferentially (131) oriented with sharp (131) and higher order Bragg reflections, with a d-spacing of 2.9163 Å. A broad, lower intensity secondary oriented (0k0) set of reflections were also observed with a d-spacing of 5.6891 Å which is close to the reported 5.6830 Å for the (020) reflection. Rocking curves measured on these samples had a very broad full width at half maximum (FWHM) $\omega$ of $7.78^{\circ }$ for the as-deposited film, indicating a poor degree of preferential orientation of the grains in these polycrystalline films. The films were annealed under vacuum to avoid oxidation of the layers at temperatures of 200, 350 and $500^{\circ }$C which was just below the reported decomposition point of the semimetal in the binary phase diagram^23^. Vacuum annealing was observed to enhance the mosaicity of the films, narrowing the rocking curves to $3.5(1)^{\circ }$ for annealing at $350\, ^{\circ }$C and $4.7(1)^{\circ }$ for annealing at $500\, ^{\circ }$C. Annealing was observed to promote the crystallinity of the secondary (0k0) oriented grains of the film increasing their intensity with increasing temperature without any corresponding shift in d-spacing. Annealing at $200\, ^{\circ }$C also resulted in the emergence of a low intensity (00l) orientation in the polycrystalline sample.

**Figure 2:**
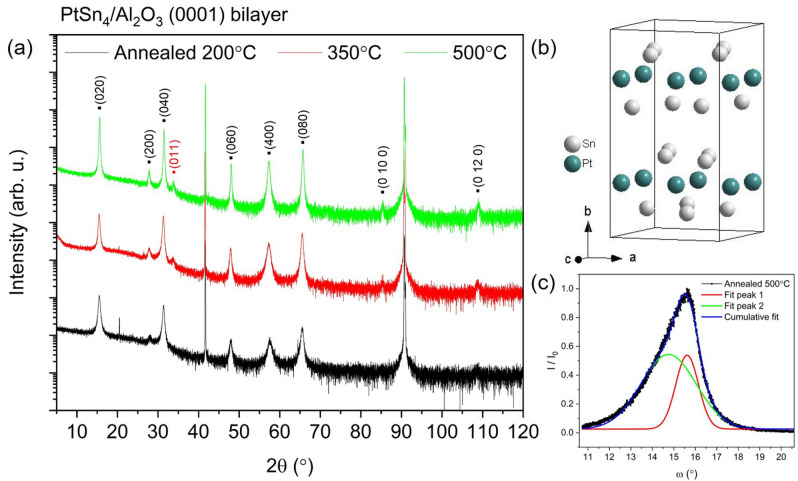
(**a**) 2$\theta$ X-ray diffraction scans of bilayer deposited $\hbox {PtSn}_{4}$ thin films grown on (0001) oriented $\hbox {Al}_{2}\hbox {O}_{3}$ substrates. The graph shows the diffraction pattern of the films which were vacuum annealed at $200^{\circ }$ (black), $350^{\circ }$ (red) and $500^{\circ }$ (black). The observed Bragg reflections are labelled on the diffractograms, the (011) reflection of the $\hbox {SnO}_{2}$ impurity phase is labelled in red. (**b**) Crystal structure of $\hbox {PtSn}_{4}$ in the Ccce space group setting. (**c**) The normalized intensity ($I/I_0$) rocking curve of the bilayer $\hbox {PtSn}_{4}$ film annealed at $500^{\circ }$ is fitted to two Gaussian peaks with their cumulative peak fit of the (040) peak shown in blue.

The Sn/Pt bilayer samples were deposited on (110) and (111) surfaces of $\hbox {SrTiO}_{3}$ and on c-cut $\hbox {Al}_{2}\hbox {O}_{3}$. X-ray diffraction patterns indicated the growth of single phase $\hbox {PtSn}_{4}$ on $\hbox {Al}_{2}\hbox {O}_{3}$ substrates as illustrated by Fig. 2a. This demonstrates that the thermodynamics of the growth process were sufficient to crystallize the targeted material with a homogeneous distribution of Pt and Sn of the intermetallic phase. $\hbox {PtSn}_{4}$ has a layered structure which crystallizes in the orthorhombic crystal system, shown in Fig. 2b, with lattice parameters a = 6.418(3) Å, b = 11.366(6) Å and c = 6.384(2) Å^30,31^. It was originally assigned the noncentrosymmetric space group Aba2 (41)^32^ however low displacement parameters enabled refinement in the higher symmetry centrosymmetric Ccce (68) space group. The structure consists of a-c planes stacked in a Sn-Pt-Sn sequence along the long b axis, where a $\cong$ c, revealing a pseudotetragonal symmetry with anisotropy in the electrical resistivity and the magnetization between the a-c plane and the b axis^8^. The as-deposited bilayer and $200 \, ^{\circ }$C annealed bilayer grown on sapphire were single phase $\hbox {PtSn}_{4}$ with dual (0k0) and (h00) orientations. The d-spacings of the $200\, ^{\circ }$C and $500\, ^{\circ }$C annealed samples were 5.6997 Å and 5.6714 Å respectively, with a shift to lower d-spacing upon heat treatment at the highest temperature. Vacuum annealing at temperatures above $200\, ^{\circ }$C resulted in the emergence of a $\hbox {SnO}_{2}$ impurity phase with a corresponding (011) Bragg reflection. The sample annealed at $200\, ^{\circ }$C exhibited a rocking curve of the (040) reflection with a FWHM $\omega$ of $1.99^{\circ }$ indicating a good degree of mosaicity but no clear in-plane epitaxial relationship with the substrate can be deduced from reciprocal spave mapping (Fig. S2). Annealing at $500\, ^{\circ }$C was observed to yield an asymmetric measured rocking curve shown in Fig. 2c for the (040) peak. The rocking curve was fitted with two cumulative Gaussian peaks with a far narrower FWHM $\omega$ of $1.06(1)^{\circ }$ and $2.57(1)^{\circ }$ respectively. This may indicate the increased presence of defects at the interface with a slight tilt due to the substrate miscut there. The as-deposited co-deposited $\hbox {PtSn}_{4}$ film grown on $\hbox {Al}_{2}\hbox {O}_{3}$ exhibited a rocking curve with a FWHM $\omega$ of $7.78(3)^{\circ }$ for comparison, demonstrating the inferiority of this growth technique.

**Figure 3:**
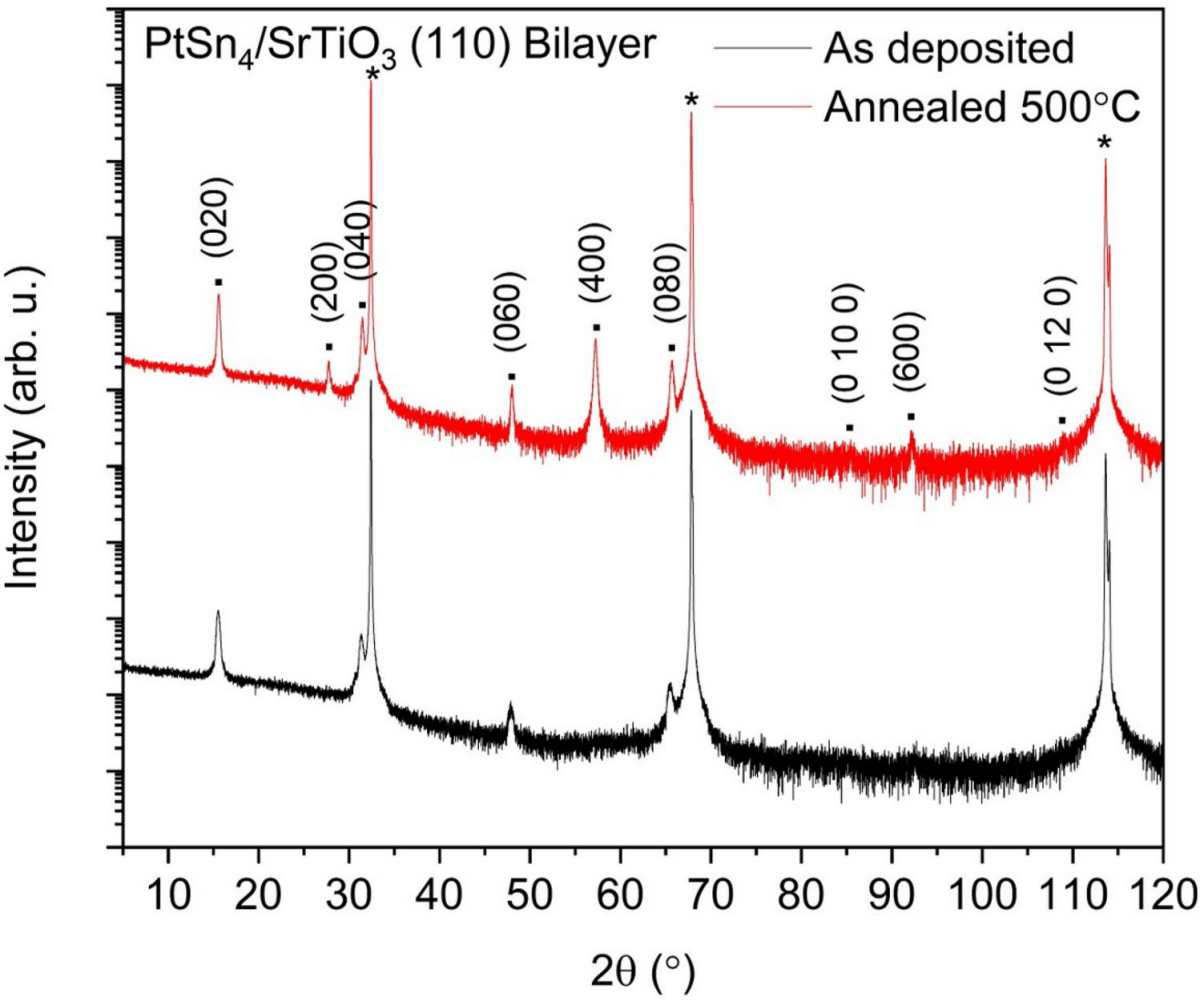
2$\theta$ X-ray diffraction scans of Pt/Sn bilayer-deposited $\hbox {PtSn}_{4}$ thin films grown on (110) oriented $\hbox {SrTiO}_{3}$ substrates. The graph shows the diffraction pattern of the as-deposited film (black) together with the film which was vacuum annealed at $500^{\circ }$ (red). The observed Bragg reflections are labelled on the diffractograms.

The as-deposited bilayer sample grown on $\hbox {SrTiO}_{3}$ (110) substrates was superior in that this was the only sample where the grains were singly-oriented along the long (0k0) axis of $\hbox {PtSn}_{4}$ as shown in Fig. 3. A d-spacing of 5.7018 Å was observed which was greater than the bulk value of 5.6830 Å in the literature and the 5.6891 Å figure which was extracted for the co-deposited $\hbox {PtSn}_{4}$ film on sapphire, this may indicate that the film is strained to the $\hbox {SrTiO}_{3}$ substrate. The crystallinity of this film was established through measurement of a rocking curve on the (020) reflection which was fitted to a Gaussian profile resulting in a FWHM $\omega$ of $3.65^{\circ }$. This peak was narrower than those of the co-deposited films indicating a relatively high degree of preferred orientation, but it was nonetheless broader than the bilayer film grown on sapphire. This may be an indication that the lattice parameters of the singly-oriented film were strained away from their bulk parameters resulting in a slight degradation of the crystalline quality. Conversely the bilayer films deposited on sapphire may consist of two distinct layers with slightly different lattice parameters. The growing overlayer which effectively grows on a $\hbox {PtSn}_{4}$ template is essentially growing on an isostructural surface with the effect of enhancing the crystallinity. Vacuum annealing the singly-oriented samples grown on $\hbox {SrTiO}_{3}$ (110) at 200, 350 and $500\, ^{\circ }$C had a detrimental effect on the oriented grains resulting in polycrystalline samples and heat treated samples indicated broad, unresolved peaks in the rocking curve measurements. The $\hbox {PtSn}_{4}$ film annealed at $500\, ^{\circ }$C was observed to have a d-spacing of 5.6790 Å, this was smaller than the as-deposited film and may indicate the temperature induces a relaxation of the strain as a degree of polycrystallinity and reorientation of the grains is introduced. The as-deposited films grown on the (111) oriented $\hbox {SrTiO}_{3}$ single crystals also resulted in polycrystalline films with splitting Bragg reflections indicating this cleavage plane was unsuited to the growth of $\hbox {PtSn}_{4}$ heterostructures.

X-ray reflectivity measurements were carried out on each of the samples indicating that the co-deposited $\hbox {PtSn}_{4}$ layers on $\hbox {Al}_{2}\hbox {O}_{3}$ were too rough to observe Kiessig fringes. These thickness-related fringes were however observed for the bilayer deposited samples grown on sapphire and (110) $\hbox {SrTiO}_{3}$ substrates and vacuum annealed at $200\, ^{\circ }$C and above, indicating reduced surface and interfacial roughnesses and island growth in these samples and confirmed the targeted thickness of 30 nm (Fig. S3) monitored using in-situ quartz balance.

The composition of the film samples was analysed using energy dispersive X-ray (EDX) analysis and XPS. The calculated atomic Pt:Sn ratios obtained from the XPS survey scan performed on a film grown on $\hbox {SrTiO}_{3}$ shows a composition of $\hbox {Pt}_{23}\hbox {Sn}_{77}$ close to the nominal stoichiometry (Fig. S4) and the EDX performed on sample grown on (0001) $\hbox {Al}_{2}\hbox {O}_{3}$ shows similar results with a good uniformity of the composition over the scanned areas (Fig. S5).

### Electrical and magnetotransport properties

**Figure 4:**
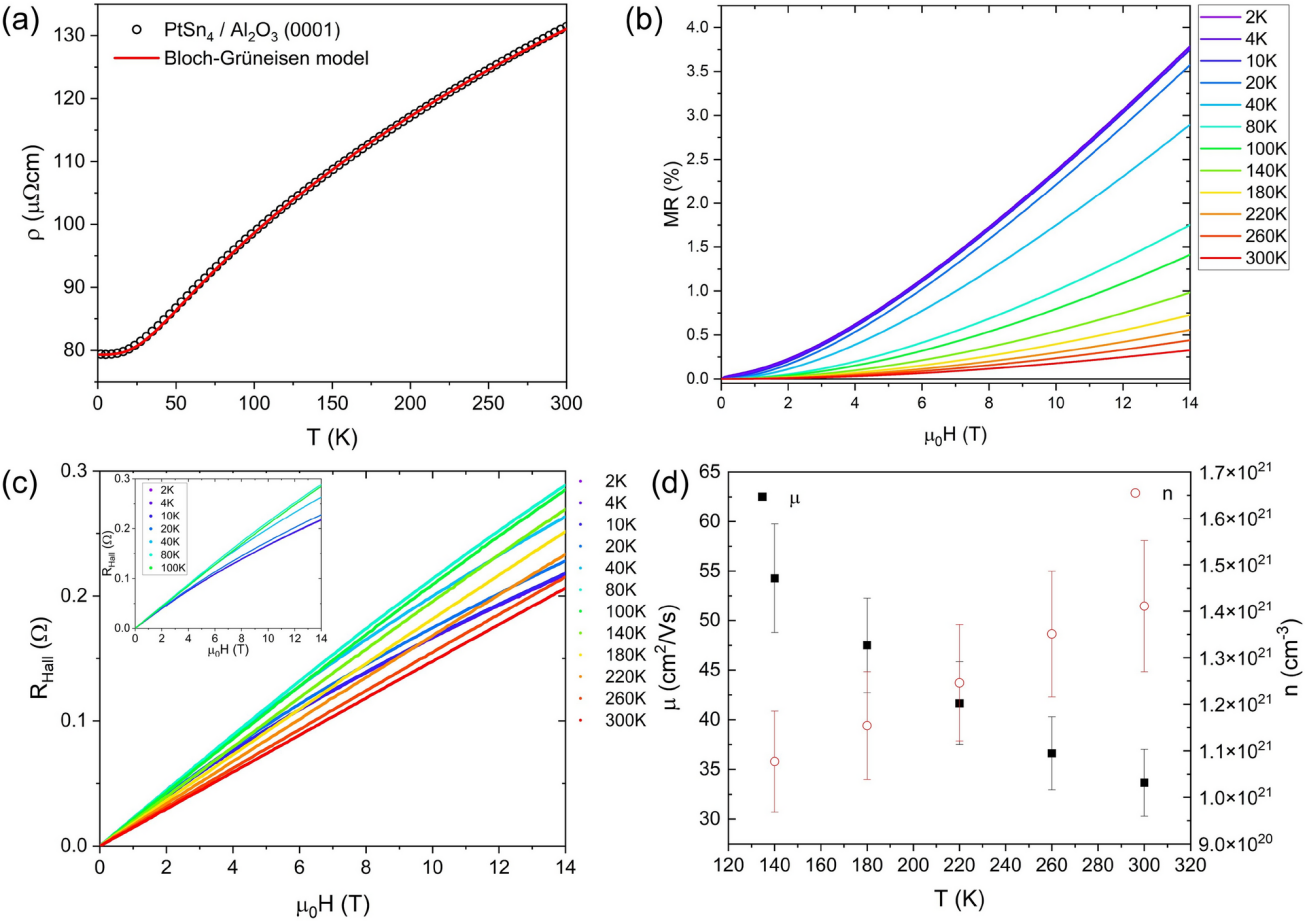
(**a**) Resistivity of bilayer deposited $\hbox {PtSn}_{4}$ thin film grown on $\hbox {Al}_{2}\hbox {O}_{3}$ as a function of temperature from 2 to 300 K with modified Bloch-Grüneisen model. (**b**) Magnetoresistance of this $\hbox {PtSn}_{4}$ film measured at a range of temperatures in applied magnetic fields up to ±14 T. (**c**) Hall resistance, $\text {R}_{Hall}$, of the $\hbox {PtSn}_{4}$ film patterned into Hall bars measured at temperatures between 2 K and 300 K in fields of up to ±14 T. The inset highlights the increasing nonlinearity in the Hall resistance emerging for temperatures below 140 K. (**d**) Effective carrier mobility and effective carrier concentration extracted from the magnetotransport measurements for temperatures of 140 K and above.

While the out-of-plane X-ray diffraction patterns indicated that the (110) $\hbox {SrTiO}_{3}$ substrate yielded the thin film with the most oriented grains, the unannealed bilayer deposited film grown on c-cut $\hbox {Al}_{2}\hbox {O}_{3}$ had the superior crystalline quality, therefore the electrical properties of this sample were examined in more details. The as-grown bilayer sample was deposited through a shadow mask to pattern Hall bars on the sample and the resistivity was measured from room temperature to 2 K, as described in the experimental methods. The electrical resistivity was observed to increase monotonically with temperature exhibiting metallic behaviour as shown in Fig. 4a. A residual resistivity value of 79.43(1) $\mu \Omega$cm was observed at 2 K, which was higher than reported values ranging from 0.013 $\mu \Omega$cm to 0.47 $\mu \Omega$cm^8,9,22,33^. A resistivity of 131.46 $\mu \Omega$cm was observed at 300 K, which was closer to a report of 42 $\mu \Omega$cm recorded at 300 K^17^, resulting in a residual resistive ratio of RRR = 1.7 which was considerably lower than the reported bulk values of between 70 and 1025^8,10,22,33^. The spread in the reported RRR values indicates the sensitive dependence of this figure on the crystalline quality. At high temperature the resistivity starts to saturate, deviating from linearity, suggesting a parallel resistor model according to Eq. (1) is appropriate^34^.

1 $$\begin{aligned} \frac{1}{\rho (T)} = \frac{1}{\rho _{el-ph}(T)} + \frac{1}{\rho _{sat}} \end{aligned}$$

The resistivity was modelled with a modified Bloch–Grüneisen fit following Eq. (2) which compares favorably with the measured data as shown in Fig. 4a, where $\rho _{el-ph}$ is the resistivity due to the electron-phonon scattering, $\rho _{sat}$ is the saturation resistivity, $\rho _{0}$ is the resistivity at 2K, $\theta _{D}$ is the Debye temperature, C is proportional to the electron-phonon coupling constant and n depends on the nature of the interaction.

2 $$\begin{aligned} \rho _{el-ph}(T) = \rho _{0} + C\left( \frac{T}{\theta _D}\right) ^{n}\int _{0}^{\frac{\theta _D}{T}} \frac{x^{n}}{(e^{x}-1)(1-e^{-x})} dx \end{aligned}$$

The model was used to extract a $\rho _{sat}$ = 2.44(1)$\times 10^{-6}$ $\Omega \, \textrm{m}$, $\rho _{0}$ = 11.75(1)$\times 10^{-7}$ $\Omega \, \textrm{m}$, and a Debye temperature $\theta _D$= 200(5) K, C = 0.225 $\times 10^{-5}$ $\Omega \textrm{m}$ with n = 3 for the electron–phonon resistivity term suggesting that *s*-*d* scattering is active^35^. This was close to the reported Debye temperature of 210 K for single crystals^8^ but lower than the 304 K measured by Mössbauer spectroscopy for $\hbox {PtSn}_{4}$ powder^36^. The room temperature resistivity of the samples deteriorated over time when the sample is left in air due to the formation of a surface oxide layer (Table S1). The temperature dependence of the sample grown on (001) $\hbox {SrTiO}_{3}$ shows similar behaviour with an upturn at low temperature which is a signature of weak localization typically observed in thin films of poorer crystalline quality (Fig. S6).

Examination of the electrical transport response of the $\hbox {PtSn}_{4}$ thin film subjected to an applied magnetic field of up to ±14 T has been measured in the range 2 K to 300 K. The symmetric component of the resistivity tensor, $\rho _{xx}$, was used to extract a positive field-dependence of the magnetoresistance at all measured temperatures as shown in Fig. 4b. The magnetoresistance increased with decreasing temperature consistent with other studies^33^ with 4% observed at 2 K, which is far below the 5 $\times 10^5$% reported in the literature for the bulk material^8,10^ (730% at 4.2 K in an applied field of 10 T^33^) this is expected in view of the high residual resistivity. The applied magnetic fields were insufficient to saturate the magnetoresistance at these temperatures. The transport parameters were extracted from the transverse antisymmetric component of the resistivity tensor which enabled the characterization of the contributing carrier types, the effective carrier concentration and the effective carrier mobility. The Hall resistance is shown in Fig. 4c, and a deviation from linearity is observed below 140 K, this indicates the presence of more than one carrier type with opposite signs, which is consistent behavior for a semimetal with two bands of opposite dispersion curvature crossing the Fermi surface. This complicates the determination of the transport parameters of the contributing carriers in this region. The data could not be fitted to a two-band model (Fig. S7) as the applied field was insufficiently high to yield an appreciable effect such as the reversal of the slope of the Hall resistance observed by Canfield et al.^8^. LuO et al. report that a multi-band model with more than three bands is required to fit the Hall conductivity in single crystals below 55 K^9^. Above 140 K the majority carriers were found to be electrons, this is consistent with the observations of Marchenkov et al. who observed majority hole carriers at 4.2 K, below the compensation point^37^. This is in contrast, however, with the results of Canfield et al. who found majority carrier hole conduction above 100 K^8^, and Luo et al. who recorded effective hole conduction above a compensation point of 30 K using a multiband model^9^. The extracted transport parameters are plotted in Fig. 4d showing the effective carrier concentration of n = 1.4(1)$\times 10^{21}$ $\hbox {cm}^{-3}$ was one order of magnitude lower than the 2.6$\times 10^{22}$ $\hbox {cm}^{-3}$ reported by Canfield et al.^8^, but close to the 6.8$\times 10^{21}$ $\hbox {cm}^{-3}$ value observed by Marchenkov et al. for the majority carrier holes at 4.2 K^33^. Effective carrier mobilities of $\mu$ = 33.6 ± 3.4 $\hbox {cm}^2\hbox {V}^{-1}\hbox {s}^{-1}$ at 300 K were found to be one order of magnitude lower than the 200 $\hbox {cm}^2\hbox {V}^{-1}\hbox {s}^{-1}$ reported by Li et al.^17^ and considerably lower than the 1950 $\hbox {cm}^{2}\hbox {V}^{-1}\hbox {s}^{-1}$ at 4.2 K reported by Perevalova et al.^33^ measured in single crystals. Comparison of the reported magnetotransport properties of single crystals with these bilayer-derived $\hbox {PtSn}_{4}$ thin films may be complicated by the influence of the layer thickness on the electronic properties of some Pt-based compounds including dichalcogenides^38,39^. Although these findings demonstrate the dramatic effect of the crystalline quality on the electrical properties, these intermetallic thin films are still semimetallic.

**Table:** 

Transport parameters	Measured	Literature
Residual resistivity (2K)	79.43(1) *μ*Ωcm	0.013–0.47 *μ*Ωcm^8,9,22,33^
Resistivity (300 K)	131.46 *μ*Ωcm	42 *μ*Ωcm^17^
RRR	1.7	70–1025^8,10,22,33^
Magnetoresistance	4% (2K)	5×10^5^%, 730%^8,10,33^
Mobility	33.6 ± 3.4 cm^2^V^−1^s^−1^	200 ﻿cm^2^V^−1^s^−1^, 1950 ﻿cm^2^V^−1^s^−1^^17,33^
Carrier density	1.4(1)×10^21^ cm^−3^	2.6×10^21^ cm^−3^﻿, 6.8×10^21^ cm^−3^﻿^8,33^

### Electronic structure and density functional theory

**Figure 5:**
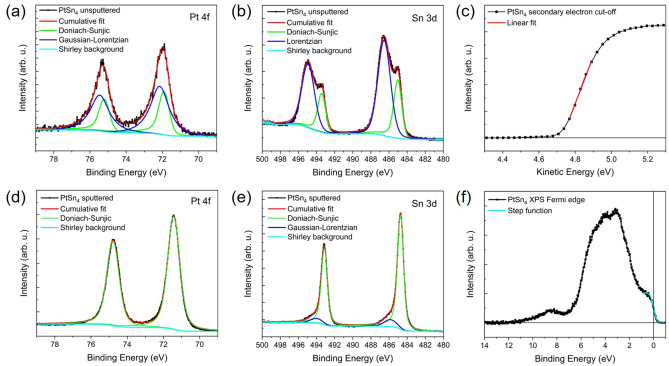
(**a**) X-ray photoemission spectrum of Pt 4*f* levels of $\hbox {PtSn}_{4}$ thin film (black) prior to sputtering showing Marquardt fit (red) of data with Doniach-Sunjic (DS) (green) and Gaussian-Lorentzian (GL) (blue) line shapes corresponding to the Pt 4$f_{5/2}$ and 4$f_{7/2}$ lines. (**b**) Core level Sn 3*d* spectrum fitted with DS (green) and Lorentzian (blue) doublets corresponding to the Sn 3$d_{3/2}$ and 3$d_{5/2}$ levels prior to sputtering. (**c**) Secondary electron cut-off energy spectrum of sputtered film as a function of kinetic energy. (**d**) Corresponding Pt 4*f* lines of $\hbox {PtSn}_{4}$ film with DS (green) line shape after sputtering to expose pristine surface. (**e**) Sn 3*d* lines fitted with DS (green) and reduced GL (blue) line shapes after sputtering. (**f**) XPS spectrum of the Fermi edge of the sputtered $\hbox {PtSn}_{4}$ film fitted with a step function for calibration of the Fermi level.

X-ray photoemission spectroscopy was carried out on the bilayer-deposited $\hbox {PtSn}_{4}$ film on $\hbox {SrTiO}_{3}$ and the data was analyzed using Casa XPS software with a Marquardt curve fitting algorithm. The objective was to examine the chemical environment of the core Pt 4*f* and Sn 3*d* levels and the Fermi edge region of this semimetal close to the Fermi level. The observation of Sn 3*d* and Pt 4*f* core level peaks confirmed the expected chemical composition in the survey spectrum. These peaks were more closely analyzed by subtracting Shirley-backgrounds and integrating the intensity of the spectra. The $\hbox {PtSn}_{4}$ film was sputtered prior to collection of the spectra to remove the oxidized layer and expose a pristine surface, the unsputtered data are shown in Fig. 5a, b, and the spectra corresponding to the sputtered film are presented in Fig. 5c–f. $\hbox {PtSn}_{4}$ is metallic with sufficiently high mobility and density of the carriers to introduce a degree of asymmetry to the core level peaks^40^, however the surface layer exhibited clear evidence of oxide components. The Pt 4*f* lines, were therefore fitted with a combined Doniach–Sunjic (DS) line shape doublet, with asymmetry parameter $\alpha$ = 0.08 and Gaussian width n = 500 corresponding to the metallic Pt, and a Gaussian-Lorentzian (GL) doublet which likely corresponds to a Pt oxide surface layer as shown in Fig. 5a for the unsputtered film. The Pt 4*f* core levels of the sputtered film confirmed the elimination of the oxide species exhibiting a single DS doublet in Fig. 5d for the sputtered film with $\alpha$ = 0.01 and n = 250. A clear enhancement of the signal was observed in the sputtered sample. The Pt 4*f* core lines of the unsputtered film revealed a doublet at 72.1 eV and 75.4 eV corresponding to the 4$f_{5/2}$ and 4$f_{7/2}$ levels of Pt metal shown in Fig. 5a. Sputtering the film resulted in a shift to lower binding energy with peaks at 71.6 and 74.9 eV as shown in Fig. 5d. It also resulted in the slight broadening of the FWHM of the metallic component from $0.67^\circ$ to $0.80^\circ$, despite a considerable increase in the spectral weight, and the simultaneous elimination of the GL peak with a FWHM of $1.21^\circ$.

The Sn 3*d* profiles were more asymmetrical than their Pt 4*f* counterparts and were therefore fitted with a set of DS peaks combined with a Lorentzian doublet as shown in Fig. 5b for the unsputtered film, and a DS doublet combined with a GL set for the sputtered film as shown in Fig. 5e. For the unsputtered sample, the lower intensity DS model doublet, with $\alpha$ = 0.065 and n = 1000, the observed peaks at 493.6 and 485.2 eV corresponded to the Sn 3$d_{3/2}$ and 3$d_{5/2}$ lines respectively of $\hbox {Sn}^0$ metal as shown in Fig. 5b. The dominant peaks were observed at higher binding energies of 495.1 and 486.7 eV and they were fitted with a Lorentzian line shape which is attributed to the non-metallic oxidized surface layer. The FWHM of the DS doublet of the unsputtered surface was $1.02^\circ$ while the Lorentzian profile was $1.64^\circ$. In the sputtered film surface the intensity of the DS doublet was observed to increase significantly whereas the peaks corresponding to the oxide component were dramatically reduced as shown in Fig. 5e. The position of both doublets were shifted toward slightly lower binding energy upon sputtering, with the DS 3$d_{3/2}$ and 3$d_{5/2}$ peaks at 493.3 and 484.9 eV and the GL lines at 494.2 and 486.0 eV respectively. The reduced spectral intensity of the GL doublet indicated a change in the chemical environment of this element which could be due to the removal of oxidized Sn species on the surface. These spectral lines compare with reported XPS studies of $\hbox {PtSn}_{4}$ single crystals^17^. The spectral positions of the high intensity 3*d* doublet were close to the reported positions for metallic Sn. The DS doublet had a narrow FWHM of $0.78^\circ$ which accounted for the greater integrated area of the peak, while the GL doublet was broader with a FWHM of $1.60^\circ$ with a much lower intensity. O 1*s* lines were also collected which were prominent in the core level spectrum of the unsputtered film and could be fitted with GL profiles centered at 532.1 and 530.6 eV with FWHM values of $1.80^\circ$ and $1.43^\circ$. This likely results from the oxidation of the exposed surface and the possible formation of an oxide of Sn. The integrated area of the O 1*s* line is sharply reduced upon sputtering, with a coincident reduction in the Lorentzian peaks of the Sn 3*d* doublet and promotion of the DS peaks, shown in Fig. 5b, e, this indicates that the oxide layer has been mostly removed.

The secondary electron cut-off energy of the $\hbox {PtSn}_{4}$ film was measured with a bias applied to the sample to avoid overlap with the analyzer. This enabled the determination of the work function of $\hbox {PtSn}_{4}$ which is widely studied for potential applications in catalysis^14–16^ particularly given the stable and robust supply of protected topological states with high carrier mobility close to the Fermi level to promote surface reaction^41^. The work function of the (010) surface of $\hbox {PtSn}_{4}$ was determined from this energy cut-off to be $\phi$ = 4.7(2) eV, a value which is greater than the 4.42 eV reported for Sn^42^, however it is less than the 5.77 eV reported for the (111) surface of Pt metal^43^. The photoemission spectra of this metallic sample were collected in the vicinity of the zero binding energy point and the Fermi edge was fitted with a step function to calibrate the Fermi level, the corrected spectrum is shown in Fig. 5f.

**Figure 6:**
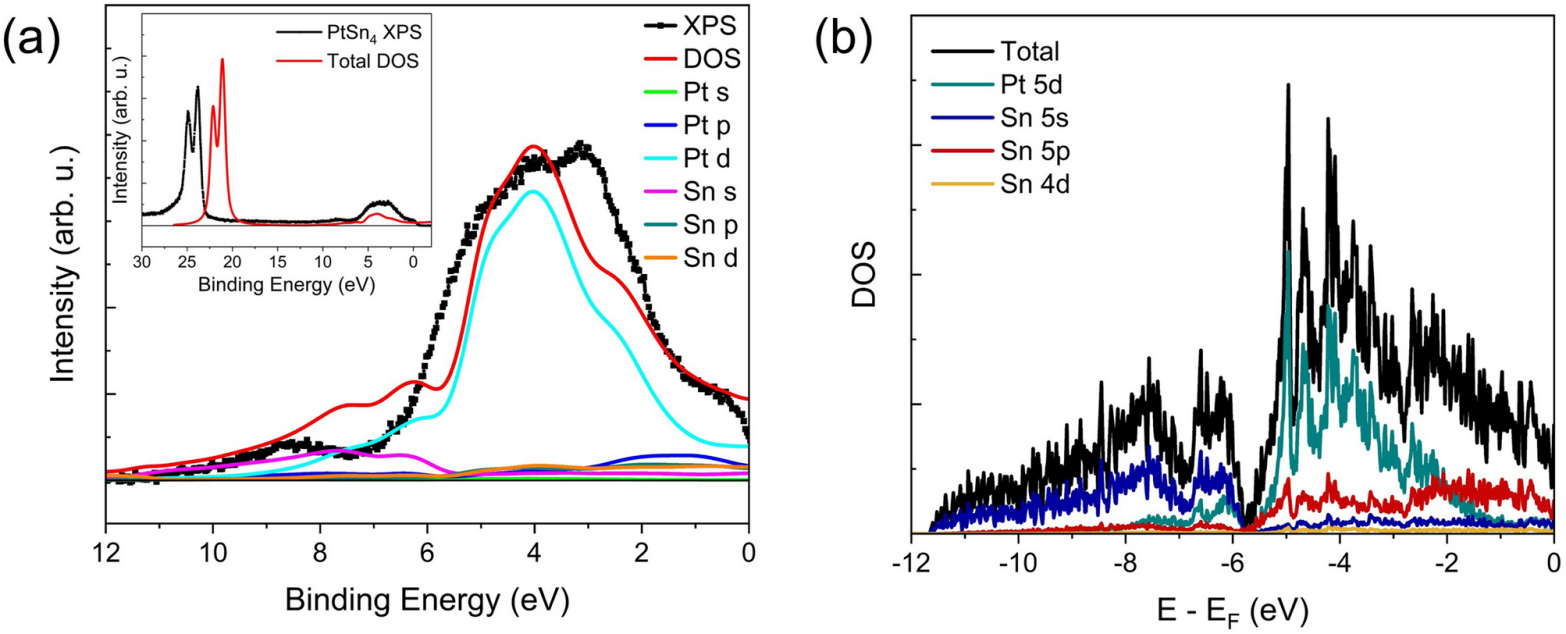
(**a**) X-ray photoemission spectrum close to the valence edge of the $\hbox {PtSn}_{4}$ film (black) compared with total (red) and partial densities of states (DOS) calculated by DFT and convolved using Galore software^29^. Inset showing the XPS spectrum (black) with total calculated DOS (red) including Sn 4*d* levels. (**b**) DFT calculation of $\hbox {PtSn}_{4}$ DOS.

The binding energy spectra of the photoelectrons below the Fermi level were collected, as shown in Fig. 6a, and the Fermi level was set to the zero of the binding energy-scale. DFT calculations were also performed for orthorhombic $\hbox {PtSn}_{4}$ to obtain the total and partial density of states (PDOS) in the Fermi edge region shown in Fig. 6b for comparison. The one-electron angular-corrected photoionization cross sections for the X-ray energy $h\nu$ = 1486.6eV have been applied to the calculated PDOS using Galore software^29^. The weighted densities of states were convolved with 0.4 eV Gaussian instrumental broadening and 0.5 eV Lorentzian lifetime broadening. The photoemission spectra have been corrected according to the calibrations and corrected for the Shirley-background, as shown in Fig. 6a, and the DFT calculations position the zero-energy point at the Fermi level. The valence band is observed to be dominated by the Pt 5*d* orbitals and to a lesser extent the Sn 5*p* and 5*s* orbitals. Comparison of the DFT calculations with the Fermi edge of the XPS spectra indicate that they map well to the spectra for the calculations. The $\hbox {PtSn}_{4}$ XPS spectrum together with the total calculated DOS over an extended energy region capture the Sn 4*d* lines indicating a shift to lower binding energy of the calculated DOS as shown in Fig. 6a inset. This DFT underestimation of *d* state semi-core level binding energies is routinely observed for particular exchange correlation functionals including PBE^44^. The *d* state underbinding may also increase the Pt 5*d* state contribution to the valence states at lower energies close to 6.3 eV while underestimating the contribution to the states at higher binding energies near 8.7 eV.

### Conclusion

The growth of oriented crystalline $\hbox {PtSn}_{4}$ thin films has been established on single crystal substrates of (110) oriented $\hbox {SrTiO}_{3}$ and c-cut $\hbox {Al}_{2}\hbox {O}_{3}$ using a Pt/Sn bilayer deposition electron beam evaporation. The greatest crystallinity was obtained for the thin films deposited on sapphire. Thermal vacuum annealing was observed to introduce tertiary orientations of the intermetallic films and an oxide impurity phase. Transport properties of the $\hbox {PtSn}_{4}$ thin film were measured including resistivity, magnetoresistance and Hall effect in a Hall bar configuration. The measured residual electrical resistivity which gives an indication of the quality of the crystal was inferior to the values reported for single crystals by between 1 and 3 orders of magnitude as expected for these polycrystalline thin films^8,11,33^. The temperature dependence of the resistivity following a Bloch-Grüneisen trend at low temperatures indicates an *s*-*d* electron-electron scattering mechanism in addition to the electron-phonon scattering. The Hall effect measurement enabled us to deduce that the majority carriers at room temperature were electrons. The nonlinearity in the Hall resistance indicates that the electrons and holes scatter differently in $\hbox {PtSn}_{4}$ as the temperature decreases, however due to the inferior crystallinity of the films in comparison to the single crystal samples we were unable to extract the majority carrier parameters at low temperatures. The carrier concentration at room temperature was only a factor of 10 reduced compared with reported values indicating that the charge carrier electrons were slightly more compensated by the holes in the thin film samples, while the effective mobility of the majority charge carriers was two orders of magnitude lower than the single crystals^8^. The magnetoresistance of the films was also far inferior to the single crystal reports indicating the strong influence of crystallinity on the electrical and magnetotransport properties of this intermetallic semimetal as demonstrated by the range of magnetoresistance values measured by Canfield et al. for their various single crystal samples^8^. The spectral study of the electronic structure of the $\hbox {PtSn}_{4}$ films indicated the presence of metallic Pt 4*f* and Sn 3*d* core lines as expected and the valence band region of the electronic spectra exhibited peaks which coincided with the Pt 5*d*, Sn 5*p*, 5*s* and 4*d* levels of the DFT calculated densities of states. Textured $\hbox {PtSn}_{4}$ films have been grown which exhibit similar electrical resistivity as a function of temperature to the bulk, however the poor crystalline quality results in much lower mobilities in the magnetotransport measurements. At low temperature there is evidence of multi-carrier transport behaviour which indicates that semimetallicity is retained. The growth of crystalline $\hbox {PtSn}_{4}$ thin films will enable the study of novel topological states in this Dirac nodal arc semimetal and the potential inclusion in frameworks for future topological quantum devices.

## Supplementary Information

Supplementary Information.

## Data Availability

The data that support the findings of this study are openly available at https://datacat.liverpool.ac.uk/id/eprint/2639.
