# Supplementary material for: Heterostructure growth, electrical transport and electronic structure of crystalline Dirac nodal arc semimetal PtSn4
Source: Sci Rep. 2024 Dec 28;14:30887. doi: 10.1038/s41598-024-81679-2 (PMC11680821; doi:10.1038/s41598-024-81679-2)
Supplement: Supplementary file 1 — Supplementary Information. [file 41598_2024_81679_MOESM1_ESM.pdf]

Supplementary Information for:

Heterostructure growth, electrical transport and electronic structure of crystalline Dirac nodal arc semimetal $PtSn_4$.

Edward L. Beynon[1], Oliver J. Barker[1], Tim D. Veal[1], Liam O'Brien[1], and Marita O'Sullivan[1,*]

[1]Department of Physics, University of Liverpool, Oxford Street, Liverpool, L69 7ZE, United Kingdom
[*]mosulliv@liverpool.ac.uk

X-ray reflectivity and diffraction

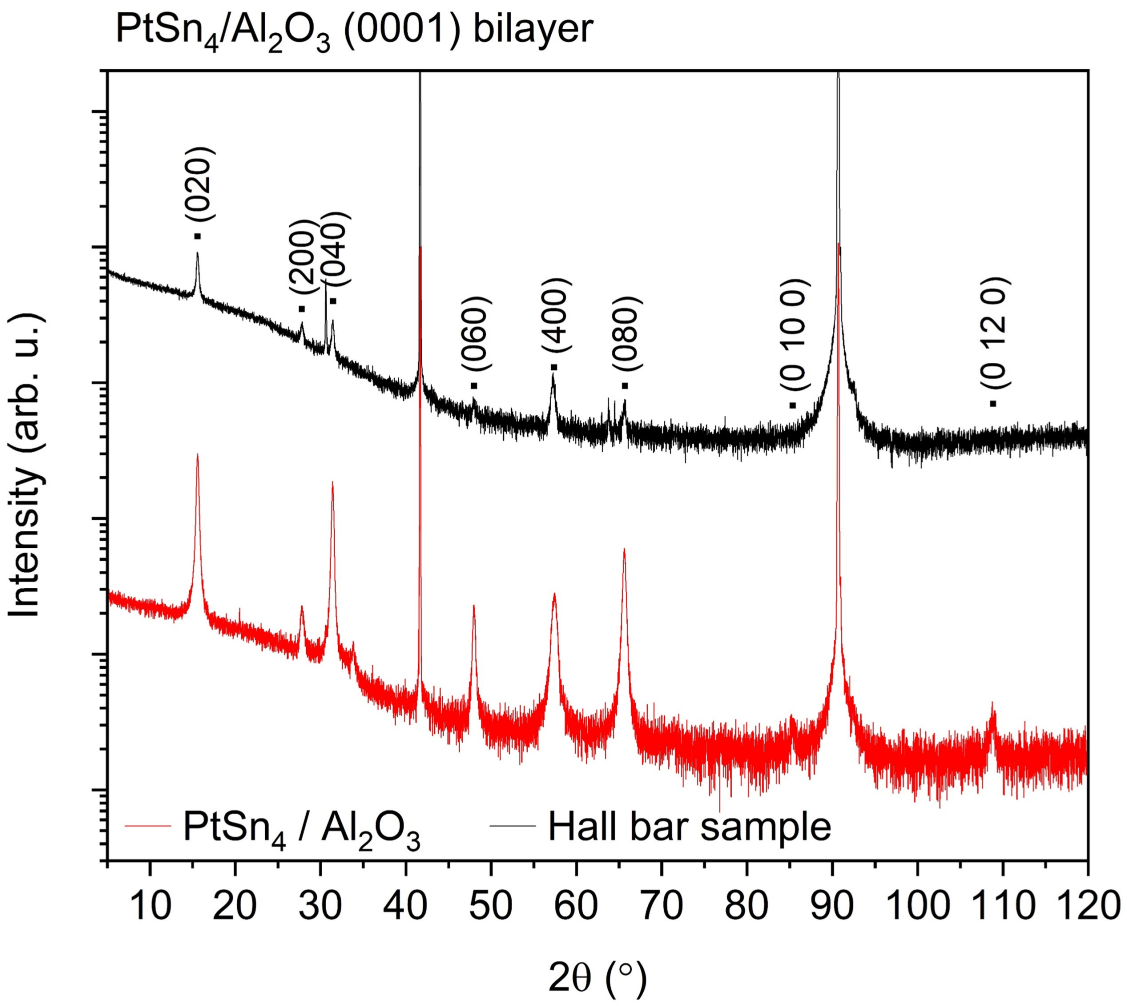

Figure S1 X-ray diffraction (XRD) pattern of the Hall bar sample (black line) displays the same orientations as the unmasked sample shown in the main text (red line), with an expected lower intensity due to the reduced sampling volume of the $PtSn_4$ Hall bars, confirming that the Hall bar pattern retains the same structural properties as the XRD studies.

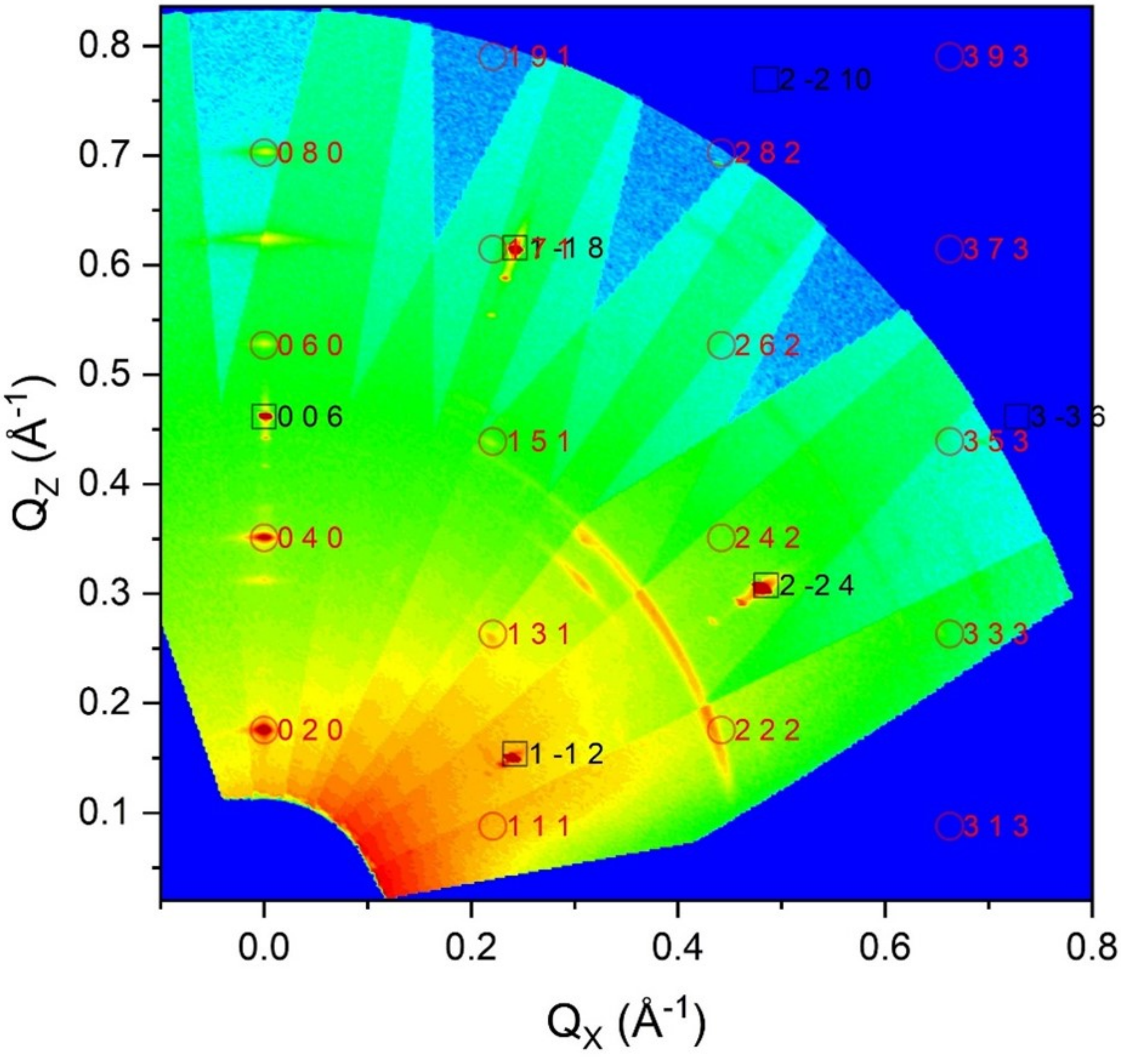

Figure S2 Wide RSM on a bilayer growth of $PtSn_4$ deposited on c-cut Al_2O_3 and annealed at 350°C. It shows that the $PtSn_4$ film (red circles) has a (010) texture with no clear in-plane epitaxial relationship with the Al_2O_3 substrate (black squares) and shows no indication of interfacial strain.

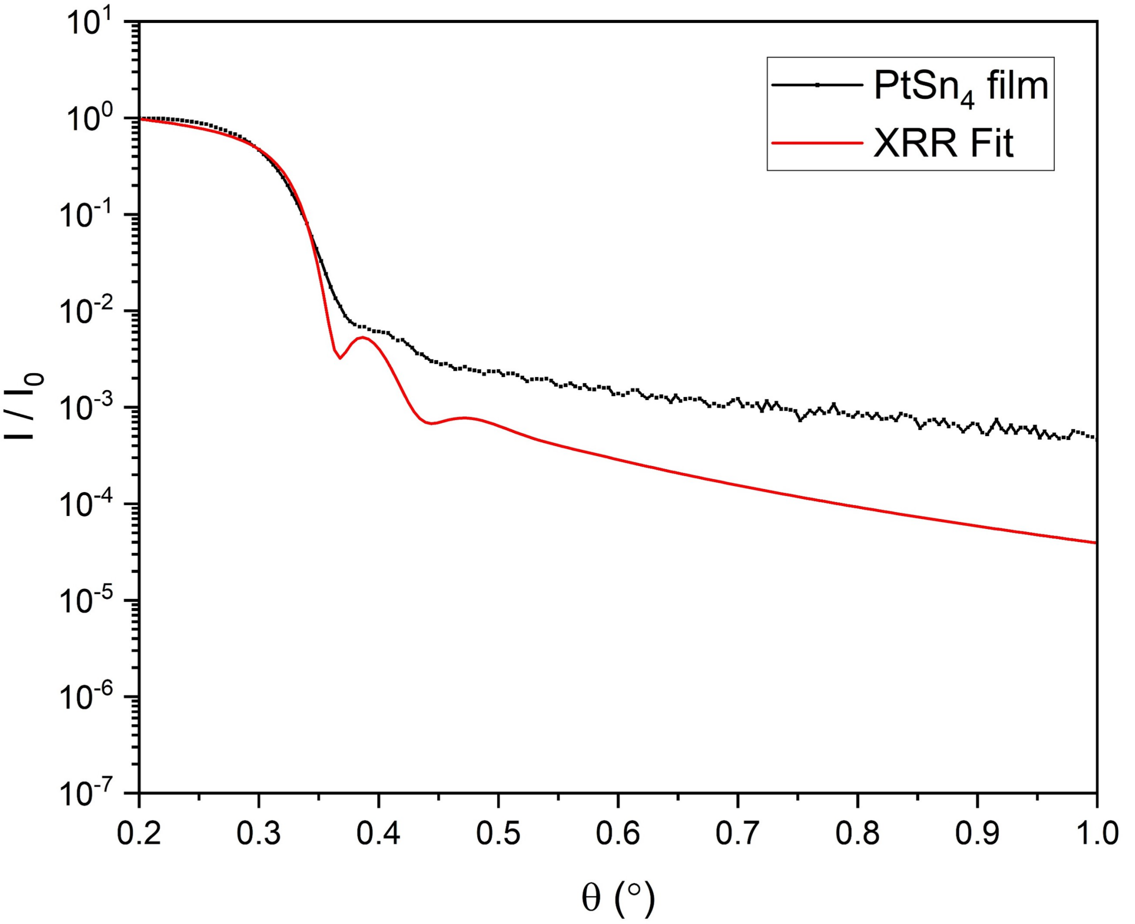

Figure S3 X-ray reflectivity scans for a bilayer growth of $PtSn_4$ thin films grown on (110) oriented $SrTiO_3$ substrates. The graph shows the experimental data (black dots), together with the simulated intensity for a 29 nm thick film.

Film composition and uniformity

XPS survey scan: composition quantification

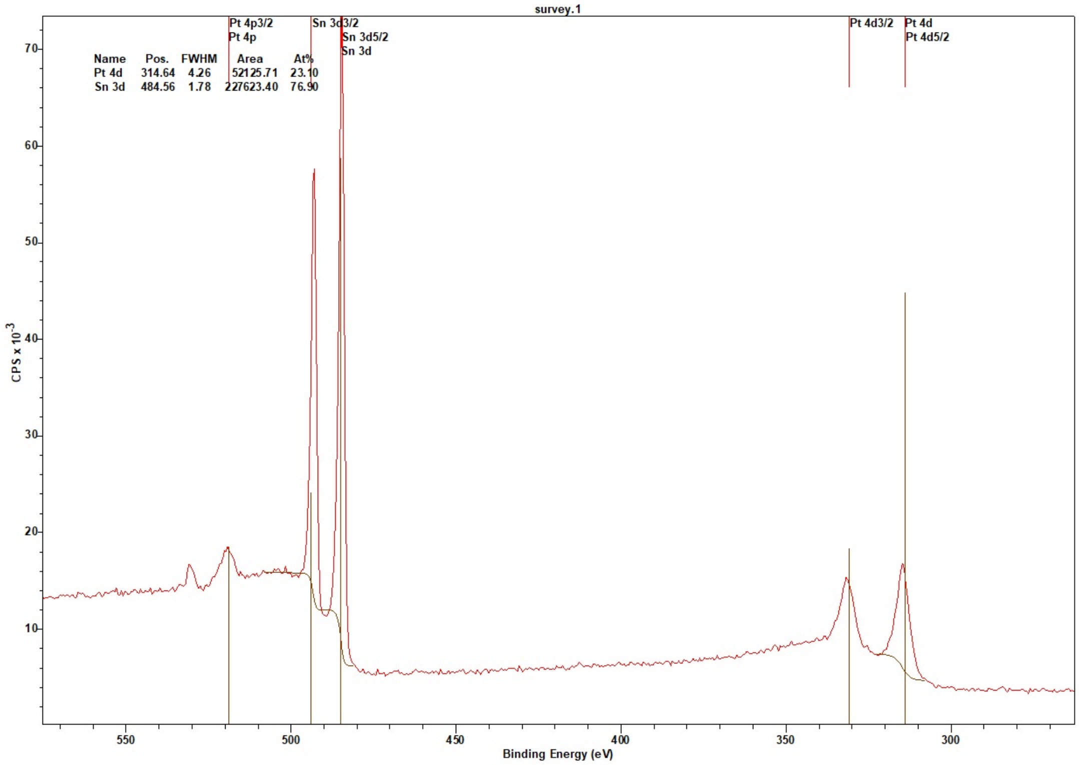

Figure S4 XPS survey scan performed on a bilayer growth of $PtSn_4$ thin films grown on (110) oriented $SrTiO_3$ substrates and annealed at 500°C (red line) and identified elemental line used for quantification (vertical lines). The elemental quantification gave an atomic composition of $Pt_{23}Sn_{77}$, suggesting a slight deficiency of Pt but close to the nominal stoichiometry.

Energy Dispersive X-ray spectroscopy: film composition uniformity

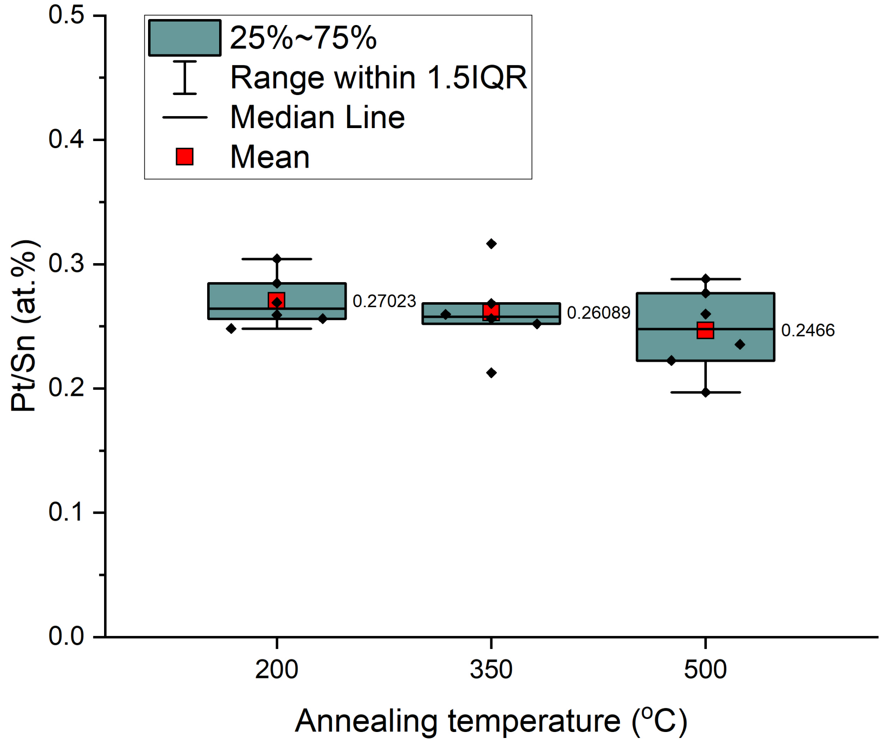

Figure S5 Box plot of the atomic ratio between Pt and Sn measured by EDX on bilayer growth of $PtSn_4$ deposited on c-cut Al_2O_3 for different annealing temperatures. Six individual spectra were collected on a regular grid over a square surface of $250\mu m^2$ (black diamonds), the mean is represented by a red square and its value is labeled at the side of the box. Overall the composition of the films is uniform and close to the expected stoichiometric ratio.

Electrical properties

Resistivity

Substrate	Annealing temperature (°C)	Room temperature resistivity (mΩ.cm)	Room temperature resistivity after 2 months in air (mΩ.cm)
Al_2O_3 (0001) Hall bar	350	0.13	27
Al_2O_3 0001	200	-	11
Al_2O_3 (0001)	500	-	12
$SrTiO_3$ (110)	200	-	11
$SrTiO_3$ (110)	350	-	27
$SrTiO_3$ (110)	500	-	$>3\times10^4$

Table S1 Electrical resistivity measured at room temperature on various bilayer growth. The resistivity increases after being left in air due to the formation of an oxide layer at the top of the films.

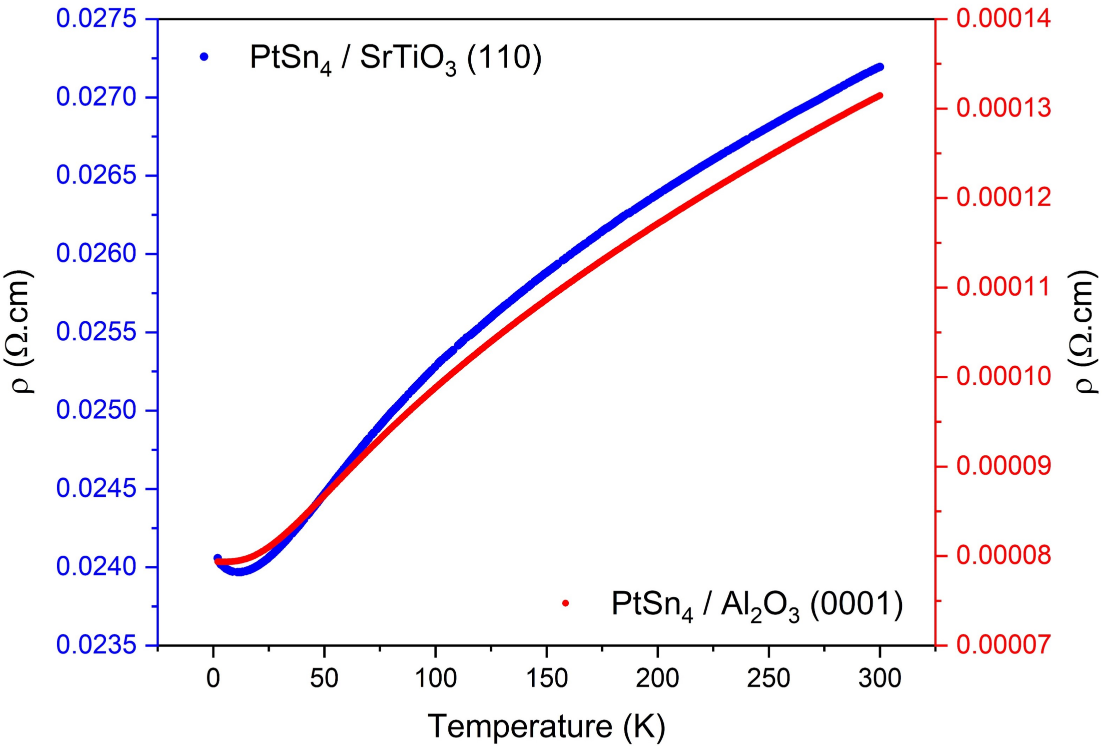

Figure S6 Electrical resistivity as a function of temperature measured two months after deposition for a bilayer growth on (110) $SrTiO_3$ (blue circles) left vertical axis compared to a bilayer growth on (0001) Al_2O_3 (red circles, right vertical axis) a week after deposition. The temperature dependence and the residual resistivity ratio are similar for both films considering the change of resistivity due to the surface oxidation. The profile of the resistivity on the film grown on $SrTiO_3$ however shows an upturn at low temperature which is a signature of weak localization typically observed in thin films of poorer crystalline quality.

Two band model analysis

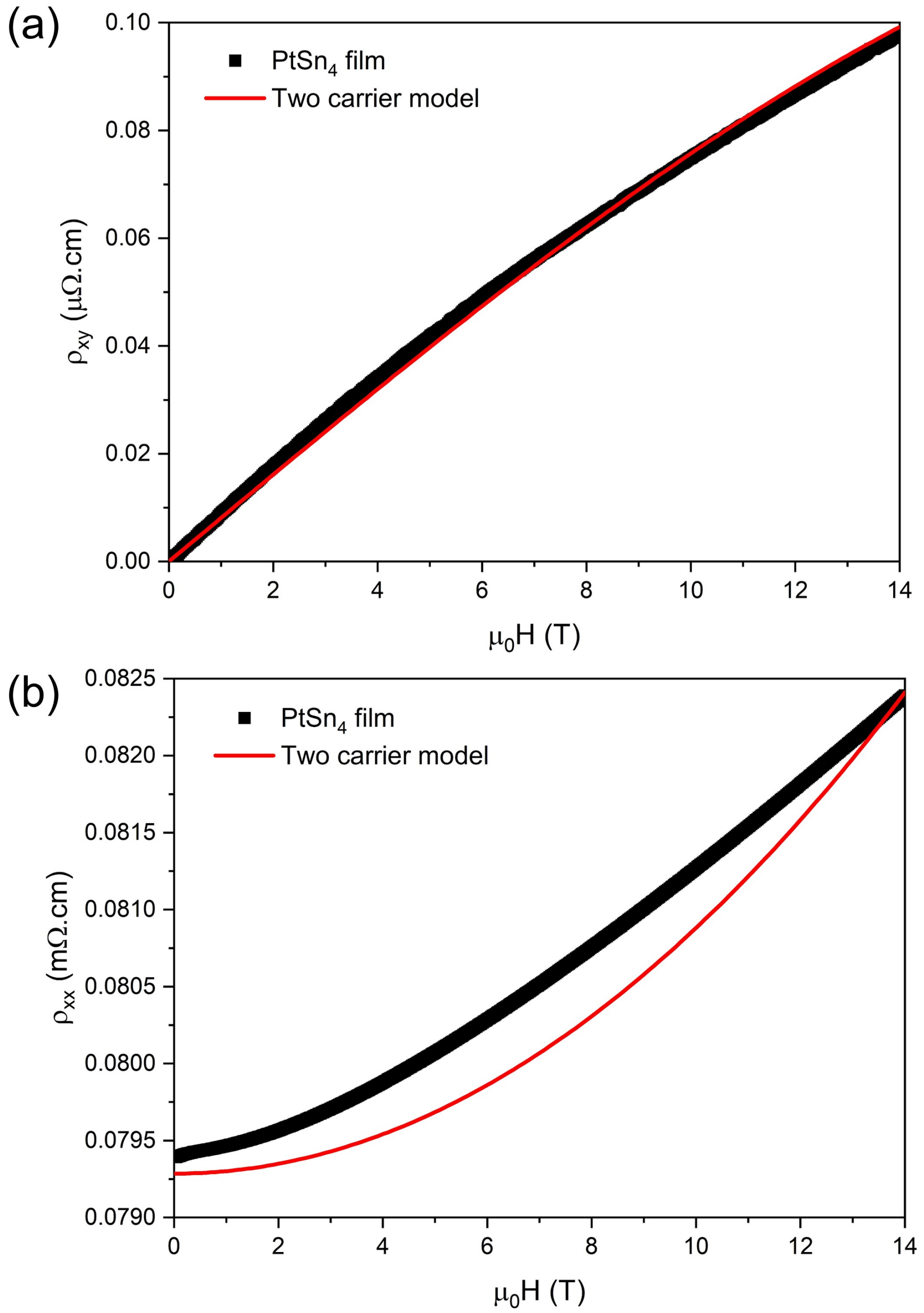

Figure S7 Two-carrier analysis of the (a) Hall effect (ρ_{xy}) and (b) magnetoresistance (ρ_{xx}) of a $PtSn_4$ film grown on Al_2O_3 (with the models shown in red) has determined majority carrier holes with a carrier concentration of 1.55×10^{21} cm^{-3} and a mobility of 120 cm^{2}V^{-1}s^{-1}, and minority carrier electrons with a carrier concentration of - 0.95×10^{21} cm^{-3} and a mobility of 165 cm^{2}V^{-1}s^{-1}.
